# Attention to Detail: The Effect of Fluoroscopic Parallax on Limb Alignment Assessment During Corrective Osteotomy

**DOI:** 10.5435/JAAOSGlobal-D-22-00289

**Published:** 2023-02-13

**Authors:** Matthew Weldon, Abraham Arenas, Alex Abraham, Layla A. Haidar, Ryan J. Warth, Alfred Mansour

**Affiliations:** From the University of Texas Health Science Center at Houston, Department of Orthopaedic Surgery, McGovern Medical School, Houston, TX.

## Abstract

The accuracy of a corrective osteotomy is dependent on many factors. One error rarely considered is using noncentered fluoroscopic imaging to assess intraoperative alignment. This study quantified the coronal alignment error produced by visual parallax per interval changes in vertical and horizontal positioning of the C-arm and alignment rod during intraoperative evaluation.

**Methods:**

Unilateral hip, ankle, and knee fluoroscopic images were obtained from a single intact cadaveric specimen. A center-center fluoroscopic image was obtained by moving the C-arm appeared in the center square of the nine-box grid. With the base of the C-arm stationary, the radiograph generator/intensifier portion of the C-arm was translated medially until the target bone appeared on the edge of the fluoroscopic image.

**Results:**

One hundred eight images were obtained. Measurement error increased by an average of 14% per 10 mm of horizontal C-arm offset. Minimal effect was seen if the obtained image was within 5 mm of the true center; however, once 55 mm of offset was reached, all experimental conditions resulted in at least 10 mm of parallax error.

**Conclusion:**

Our results demonstrate that small variations in C-arm positioning can create statistically significant inaccuracies when assessing limb alignment using intraoperative fluoroscopy.

Distal femoral and proximal tibial osteotomies are routinely done to alter limb alignment in the setting of symptomatic limb deformity. Both gradual correction (using ring external fixation) and acute correction (using internal fixation) are effective techniques.^1,2^ Long alignment radiographs are typically used to calculate the magnitude of correction, regardless of the chosen method, although intraoperative correlation aids surgeons in confirming the final correction.

In the setting of acute correction, preoperative templating to determine the magnitude of the correction and patient-specific instrumentation have become common tools to improve osteotomy accuracy.^3-5^ Despite these advances, the only intraoperative assessment of the mechanical axis is done using an alignment rod and fluoroscopic imaging of the hip, knee, and ankle. The accuracy of this intraoperative assessment is dependent on accurate projection of the intraoperative alignment rod on fluoroscopic images.

The concept of parallax is understood by reviewing the physics of fluoroscopic image acquisition. Although rarely considered, inaccurate positioning of the C-arm resulting in off-center fluoroscopic projection of the target structure may produce fluoroscopic parallax, misguided alignment evaluation, and ultimately an inaccurate osteotomy correction. This fluoroscopic parallax has seldom been studied in light of current orthopaedic practice. The purpose of this study was to quantify the coronal alignment error produced by fluoroscopic parallax per interval changes in vertical and horizontal positioning of the C-arm and alignment rod during intraoperative evaluation. We hypothesized that increasing the mediolateral distance between the center of the radiograph beam and the center of the target osteotomy sites (by adjustment of the horizontal C-arm position) would increase the degree of error between true and perceived alignment rod positioning. We also hypothesized that increasing the vertical distance between the skin surface and the C-arm detector (by adjustment of the vertical C-arm position) would increase the sensitivity of mediolateral distance measurements with changes in horizontal C-arm positioning.

## Methods

Unilateral hip, ankle, and knee fluoroscopic images were obtained from a single intact cadaveric specimen. The cadaveric specimen was placed in the supine position with the anterior superior iliac spine, patella, and toes facing upward and secured in position to control rotation and limb alignment. A nine-section orientation grid with equal dimensions was taped on the fluoroscopy monitor to guide appropriate image centering in the horizontal and vertical axes. An alignment rod was secured at the predicted centered position at the hip and ankle according to standard clinical procedures for a distal femoral or high tibial osteotomy using the absolute center of the fluoroscopic image and center of the joint as the zero point for calculation.

A center-center fluoroscopic image (true center) was obtained by positioning the C-arm such that the center of the hip and overlying alignment rod appeared in the center square of the nine-box grid. The C-arm used was the GE-OEC 9800 Plus. With the base of the C-arm stationary, the radiograph generator/intensifier portion of the C-arm was pushed medially until the target bone appeared on the edge of the fluoroscopic image. Images were obtained in 2-cm increments moving medial to lateral until the hip was on the opposite edge of the fluoroscopic image (Figure 2). The C-arm boom had calibrated markings for each centimeter to allow for precise adjustment. This procedure was repeated after changing the distance between the alignment rod and the skin. This same protocol was followed at the knee and ankle. Measurements (in millimeters) were then obtained from the saved images using a radiographic ruler to calculate the distance from the center of the joint to the center of the rod (center of the bone [Cb] to center of the rod [Cr]) as observed in the varied positions for hip and ankle calculations. Because the position of the rod at the level of the knee joint was dependent on the mechanical alignment of the cadaveric specimen, the measurements at the knee were obtained in relation to the rod position in a centered image at the level of the knee instead of the center of the joint.

Statistical analyses were conducted (linear regression after log transformation of measured distances) to assess the degree of measurement error in reference to the centered image per 1-mm unit of horizontal C-arm offset. Data are presented as the change in perceived distance per incremental change in horizontal and vertical C-arm positioning. *P* values less than 0.05 represent statistical significance. All Student *t*-test statistical analyses were conducted using a combination of MS Excel 2016 and SPSS version 23 (Armonk, NY).

## Results

**Figure 1:**
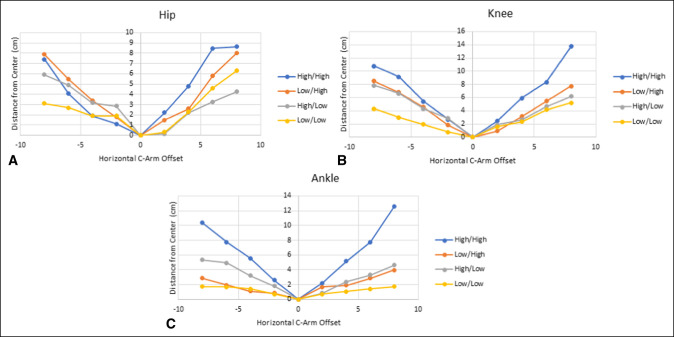
**A–C**, Graphs showing predicted measurement error in projected alignment rod positioning in millimeters per unit change in C-arm horizontal offset. The four combinations of rod and C-arm height from the specimen are individually represented.

One hundred eight images were obtained (n = 36 for the hip, knee, and ankle, respectively). Log-linear regression was used to assess the degree of measurement error per 1-mm unit of horizontal C-arm offset away from the target structure (Cb to Cr) according to the rod height above the surface of the skin (low versus high). The measurement error increased by an average of 14% per 10 mm of horizontal C-arm offset. Figure 1 depicts the error at each joint.

**Figure 2:**
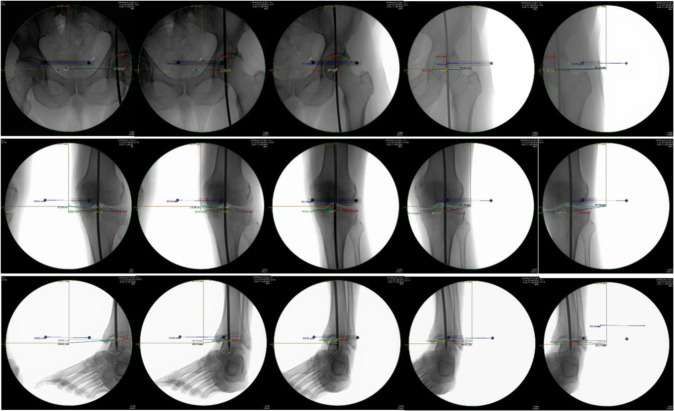
Radiographic demonstration of parallax and “movement” of the alignment rod produced by changing the position of the C-arm relative to the center of the imaged hip, knee, and ankle joints. The “true position” is the centered image of the hip, knee, and ankle that demonstrates the alignment rod centered over the joint. Moving the C-arm medially or laterally causes an apparent shift in position of the rod relative to the joint, ie, parallax.

Minimal effect was seen if the obtained image was within 5 mm of the true center; however, once 55 mm of offset was reached, all experimental conditions resulted in at least 10 mm of parallax error. (Figure 1, A–C). Rod positioning (high or low) and C-arm positioning (high or low) seemed to mildly affect distance measurements between the center of the bone and the center of the rod (Cb-Cr distance); however, the magnitude of this change is minimal compared with the effect of obtaining imaging in an eccentric position. Figure 2 demonstrates the changes in visualized rod position at the hip and knee as the C-arm is moved from the medial to lateral position.

## Discussion

**Figure 3:**
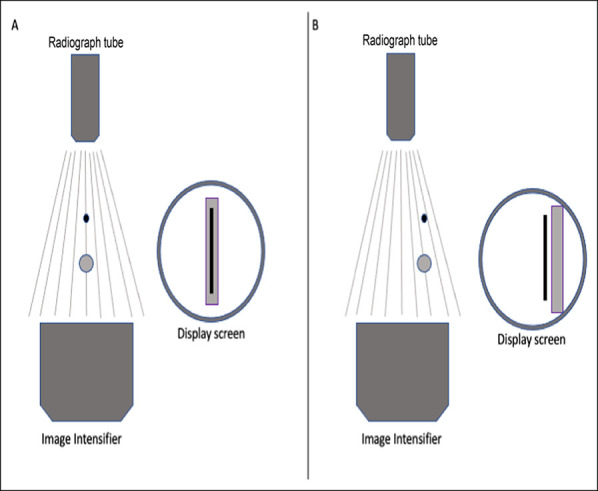
Schematic diagrams demonstrating the effect of parallax on the projection of two objects overlapping on different vertical planes. When the objects are imaged in the center of the C-arm, they project overlapped onto the display screen. When the objects are imaged off-centered, they project offset despite actually remaining overlapped in their true position.

Fluoroscopic parallax is present to some degree every time fluoroscopic imaging is used to evaluate structures placed at different vertical planes within the imaging field of view. This inherent projection anomaly is due to the divergent radiograph beam produced by the radiograph tube. When the objects are aligned in the central portion of the radiograph tube, the beam is exactly perpendicular to the objects and projects them without distortion onto the center of the image intensifier. When objects are evaluated off-centered, the divergent radiograph beams pass through the objects in nonperpendicular directions and create parallax between the two objects on the intensifier. The concept of parallax in this scenario is demonstrated in Figure 3.

Although this concept is well-discussed in the radiology literature, many surgeons do not discuss or appreciate the effect of parallax in the clinical setting. Previous studies allude to it and discuss complex formulas to minimize parallax using automated C-arms or patient tables that move in tandem with the C-arm to account for parallax when stitching images together. However, they do not discuss parallax observed when different structures are placed at different horizontal and vertical levels, such as a marker placed on the skin to evaluate osteotomies.

Keating et al^6^ reviewed the concepts of parallax and several related fluoroscopic distortions, namely pincushion distortion and S distortion. They explained that next-generation C-arms that use flat-panel detectors may minimize the distortion-related issues of standard image-intensified fluoroscopy. Similar to our study, they conclude that using well-centered images minimize the effects of parallax and fluoroscopic distortions.

Petilon et al^7^ demonstrated this concept evaluating lumbar disk arthroplasty and showed the effect of parallax when using the spinous process in relation to the center of the vertebral body. Variations as small as 1° to 3° and 1 to 3 cm produced enough parallax to misguide the placement of the disk in the central portion of the vertebral body. This was amplified because these structures are not located at the same level anatomically, similar to the alignment rod placed over the joint during osteotomy evaluation.

This well-known problem is less evident when conducting gradual correction with a ringed external fixator because intraoperative correction does not need to be precise, and gradual correction can be fine-tuned based on limb alignment assessment using full-length radiographic imaging. Postprocessing alignment can be calculated with digital imaging software tools that are not subject to parallax because the tools are placed on the postprocessed images.

Our data demonstrate that a well-centered image within 5 mm of the true center of the C-arm produces <2% horizontal parallax. The finding that at least 10 mm of horizontal measurement error (parallax) can be produced with only 55 mm of eccentric positioning of the C-arm leads us to conclude that much of the measurement error in intraoperative osteotomy positioning may be because of the influence of parallax created by a poorly positioned C-arm. 10 mm of C-arm offset parallax is enough to cause the true mechanical axis to appear to remain in the “offloaded” compartment instead of neutral or shift further into the loaded compartment (Figure 2). Previous studies have shown that the survivorship of high tibial osteotomies is affected by leaving residual deformity, and therefore, understanding the concept and consequences of C-arm malpositioning cannot be overstated.^8,9^

There are several limitations to our study. We chose to use a single cadaveric lower limb with a normal lower exterior alignment for all measurements to serve as an internal control so that the only variable would be C-arm and rod positioning. A single specimen to evaluate parallax in a spine model has been previously published.^7^ Additional specimens with more baseline deformity may produce different data; however, our specimen had nearly normal mechanical alignment and, therefore, represented the postosteotomy corrected alignment fairly closely. Additional variations could be postulated based on patient body habitus and therefore soft-tissue envelope, altering distance between the rod and the bone. We demonstrated that the height changes have only a mild effect (for hip and ankle) when compared with an eccentrically positioned C-arm. Magnification error also has to be taken into consideration because it has an increased effect on the knee when using Cb-Cr as the center-center point. We also only evaluated the mediolateral direction and did not study the effect of malpositioning the C-arm in the craniocaudal direction. There may be a similar effect in malpositioning in any outward radial direction; thus, we advocate for central positioning of the object of interest to eliminate any distortion or parallax.

## Conclusion

Our results demonstrate that small variations in horizontal C-arm positioning can create statistically significant inaccuracies when assessing limb alignment using intraoperative fluoroscopy. Our results confirm that accurately centered imaging of the alignment rod within 5 mm of the true image center at the joint level is critical to minimize error when using fluoroscopic intraoperative assessment of limb alignment. Surgeons should understand the implications of horizontal parallax during fluoroscopic evaluation and make conscious efforts to minimize its effect by critically placing the target structure in the true center of the fluoroscopic imaging screen.
